# Origin recognition complex 6 overexpression promotes growth of glioma cells

**DOI:** 10.1038/s41419-024-06764-w

**Published:** 2024-07-06

**Authors:** Wen-lei Yang, Wei-feng Zhang, Yin Wang, Yue Lou, Yu Cai, Jun Zhu

**Affiliations:** 1 Department of Neurosurgery, Ruijin Hospital, Shanghai Jiao Tong University School of Medicine, Shanghai, China; 2 Department of Radiotherapy and Oncology, Affiliated Kunshan Hospital of Jiangsu University, Kunshan, China; 3 School of Biomedical Engineering and Med-X Research Institute, Shanghai Jiao Tong University, Shanghai, China

**Keywords:** Targeted therapies, CNS cancer

## Abstract

The discovery of novel oncotargets for glioma is of immense significance. We here explored the expression patterns, biological functions, and underlying mechanisms associated with ORC6 (origin recognition complex 6) in glioma. Through the bioinformatics analyses, we found a significant increase in *ORC6* expression within human glioma tissues, correlating with poorer overall survival, higher tumor grade, and wild-type isocitrate dehydrogenase status. Additionally, ORC6 overexpression is detected in glioma tissues obtained from locally-treated patients and across various primary/established glioma cells. Further bioinformatics scrutiny revealed that genes co-expressed with *ORC6* are enriched in multiple signaling cascades linked to cancer. In primary and immortalized (A172) glioma cells, depleting ORC6 using specific shRNA or Cas9-sgRNA knockout (KO) significantly decreased cell viability and proliferation, disrupted cell cycle progression and mobility, and triggered apoptosis. Conversely, enhancing ORC6 expression via a lentiviral construct augmented malignant behaviors in human glioma cells. ORC6 emerged as a crucial regulator for the expression of key oncogenic genes, including *Cyclin A2*, *Cyclin B2*, and *DNA topoisomerase II* (*TOP2A*), within glioma cells. Silencing or KO of ORC6 reduced the mRNA and protein levels of these genes, while overexpression of ORC6 increased their expression in primary glioma cells. Bioinformatics analyses further identified RBPJ as a potential transcription factor of *ORC6*. RBPJ shRNA decreased ORC6 expression in primary glioma cells, while its overexpression increased it. Additionally, significantly enhanced binding between the RBPJ protein and the proposed ORC6 promoter region was detected in glioma tissues and cells. In vivo experiments demonstrated a significant reduction in the growth of patient-derived glioma xenografts in the mouse brain subsequent to ORC6 KO. ORC6 depletion, inhibited proliferation, decreased expression of Cyclin A2/B2/TOP2A, and increased apoptosis were detected within these ORC6 KO intracranial glioma xenografts. Altogether, RBPJ-driven ORC6 overexpression promotes glioma cell growth, underscoring its significance as a promising therapeutic target.

## Introduction

Glioma is a heterogeneous spectrum of tumors originating from glial cells [1–3]. It encompasses a spectrum of astrocytomas, oligodendrogliomas, ependymomas, and mixed gliomas, each necessitating distinct treatments [1–3]. Graded from I to IV based on aggressiveness, glioma poses significant challenges due to their infiltrative nature and resistance to current treatments [1–3]. Therapeutic approaches, typically including a combination of surgery, radiation, and chemotherapy (mainly temozolomide), depend on factors such as tumor location, histology, genetic profile, and the patient’s overall health [1, 4–7]. The treatment for glioblastoma (GBM) is especially challenging, with limited treatment success, spurring research into innovative therapies such as targeted molecular treatments, immunotherapy, and advanced drug delivery systems [1, 4–7].

Targeted therapy in glioma employs tailored treatments toward distinct molecular alterations or pathways pivotal in tumor proliferation and progression [1, 4, 6–8]. Strategies include agents addressing aberrations including EGFR amplification [9, 10], VEGFR overexpression/overactivation [11], and mutations in *isocitrate dehydrogenase 1/2* (*IDH1/2*) [12]. Immunotherapeutic agents including PD-1/PD-L1 antibodies/inhibitors aim at boosting the immune system’s response against glioma cells [13]. Yet, challenges persist due to glioma heterogeneity, adaptive resistance mechanisms, and the formidable blood-brain barrier [9, 12]. The discovery of novel oncotargets for glioma is of immense significance [9, 12].

Origin Recognition Complex (ORC) is a multi-subunit protein assembly in eukaryotic cells, pivotal in the initiation of DNA replication [14, 15]. Comprising six subunits (ORC1-ORC6), ORC orchestrates the identification and binding to specific DNA sequences of replication origins, instigating the assembly of the pre-replication complex [14, 15]. By serving as a scaffold, ORC recruits essential components necessary for the initiation and progression of DNA replication, ensuring accurate duplication of DNA during cell division [14, 15]. Perturbations of ORC may lead to disturbances in DNA replication and genomic stability, potentially fueling the onset and advancement of cancer. Within ORC, ORC6 is crucial for recognizing precise DNA sequences of replication origins [16–18]. Its role involves orchestrating the assembly of proteins essential for precise DNA duplication during cell division [14, 19–21]. Aberrations in ORC6 expression or functionality have been associated with various cancer types, contributing to increased cell proliferation and potentially influencing poor prognosis in certain malignancies such as breast, lung, colorectal, and hepatocellular carcinomas [22–26]. In the present study, we explored the expression patterns, biological functions, and underlying mechanisms of ORC6 in glioma.

## Material and methods

### Reagents and antibodies

Thermo-Fisher Invitrogen (Shanghai, China) supplied all fluorescence probes, including TUNEL, DAPI (4’,6-diamidino-2-phenylindole), EdU, Annexin V, and propidium iodide (PI). All antibodies were purchased from Cellular Signaling Tech (Danvers, MA) and Abcam (Cambridge, United Kingdom). The verified mRNA primers and siRNAs were from Genechem (Shanghai, China). Hyclone (Logan, UT) supplied fetal bovine serum (FBS), high-glucose medium, and antibiotics. Sigma-Aldrich (St. Louis, Mo) provided caspase inhibitors, puromycin, polybrene, and other chemicals.

### Human tissues and cells

A total of twenty (*n* = 20) primary patients provided written-informed consent and donated high-grade glioma (HGG) tissues and adjacent brain tissues, as described in earlier publications [27–30]. Fresh tissue lysates were analyzed [28–30]. Moreover, Dr. Cao from Soochow University provided the primary human glioma cells from three patients (“P1” “P2” and “P3”), with written-informed consent, and primary human astrocytes (“Astrocytes1/2”) from patients “P1” and “P2” [15, 16, 18, 19]. The immortalized glioma cell line (A172) was also provided by Dr. Cao [29, 31–33]. All procedures involving primary human tissues and cells were conducted in accordance with the Declaration of Helsinki and approved by the Ethics Board of Shanghai Jiao Tong University.

### Western blotting

Upon extraction of total proteins, quantification was performed using the BCA protein assay kit (Thermo Fisher Scientific). An equal quantity of protein underwent separation via 10-15% SDS-polyacrylamide gel electrophoresis (SDS-PAGE) and subsequent transfer to a polyvinylidene fluoride (PVDF) blot at 4 °C. The protein was then subjected to incubation with primary and secondary antibodies successively, held at 4 °C. The blotting signaling was observed using the Amersham ECL plus Western Blot system. Figure S1 listed all uncropped blotting images.

### RNA isolation and qPCR

TRIzol was used to extract total RNA from cellular and tissue lysates. RNA concentration and quality were assessed using the Nanodrop 2000 spectrophotometer. Reverse transcription was performed using the Promega M kit to generate cDNA. Subsequently, qPCR was conducted using the SYBR Green PCR kit (Thermo Fisher Scientific), and quantification was carried out via the 2^-ΔΔ^Cq method, with *GAPDH* serving as the internal control.

### shRNA

Genechem (Shanghai, China) provided lentivirus (GV369 construct) encoding two distinct ORC6 shRNAs (“shORC6-s1”/“shORC6-s2 “), as well as lentivirus (GV369 construct) encoding two distinct RBPJ (recombination signal binding protein for immunoglobulin kappa J region) shRNAs (“shRBPJ-Sq1”/“shRBPJ-Sq2 “). Cells were cultured in complete medium with polybrene and were exposed to the lentivirus (at MOI = 12) for 48 h. Subsequently, cells were sustained in medium containing puromycin for an additional eight-ten days to establish stable cells, where ORC6/RBPJ expression was consistently assessed through qRT-PCR and Western blotting analyses.

### Gene overexpression

Genechem (Shanghai, China) provided lentivirus (GV369 construct) containing the hORC6-expressing sequence or the hRBPJ-expressing sequence. Cells were cultured in complete medium supplemented with polybrene and infected with the ORC6/RBPJ-expressing lentivirus (at MOI = 12) for 48 h. Post-infection, cells were maintained in puromycin-containing medium for another nine days to establish stable cells. Verification of ORC6/RBPJ expression was conducted using qRT-PCR and Western blotting analyses.

### ORC6 knockout (KO)

ORC6 KO was achieved by integrating the sequence encoding the small-guide RNA (sgRNA) targeting *hORC6* into the lenti-CRISPR/Cas9-KO-puro construct (provided by Dr. Cao [31–33]). Glioma cells expressing Cas9, also from Dr. Cao [31–33], were transfected with the lenti-CRISPR/Cas9-ORC6-KO construct. Subsequently, the cells were distributed into 96-well plates and cultured in a medium containing puromycin to facilitate the selection of several stable colonies. The expression levels of ORC6 in these colonies were assessed using Western blotting. Following this, the cell clone exhibiting the most substantial downregulation of ORC6 protein was selected for further use. While there was a notable decrease in ORC6 levels, the data did not demonstrate the complete elimination of the ORC6 protein. Control cells were transduced with the lenti-CRISPR/Cas9-KO-puro control construct containing non-sense sgRNA (“Cas9-C”).

### Cell counting kit-8 (CCK-8)

Cells with the specified genetic treatments were first placed into 96-well plates at a density of 4 × 10^3^ cells per well and then cultured for designated durations. Subsequently, the CCK-8 solution was added into each well and allowed to incubate for 2 h. The optical density (OD) value of the CCK-8 in each well was measured at 450 nm.

### Nuclear TUNEL/EdU staining

Cells with the specified treatments were seeded onto coverslips in 24-well plates and cultured for designated duration. Cells then underwent fixation with 4% formaldehyde-PBS for 12 min at room temperature, succeeded by permeabilization using 0.2% Triton X-100 in PBS for an additional 8 min. TUNEL, EdU or DAPI dyes were employed to stain cell nuclei. Fluorescent images were taken from five random views utilizing a Nikon fluorescence microscope (Nikon Corporation, Japan).

### “Transwell” assays

“Transwell” chambers (Corning) were utilized and 100 μL of cell suspension containing 12, 000 cells was dispensed into individual chamber. The lower chamber contained 600 μL of medium with 12% FBS. After a 24 h incubation, the medium was removed, and non-migrated cells were eliminated using a cotton swab. To stain the migrated cells, a 300 μL staining solution was added for 10 min. Subsequently, the chamber underwent several rinses with PBS and cells were photographed. For invasion assays, a thin layer of Matrigel (100 µg/cm², Sigma) was applied to the “Transwell” inserts.

### Fluorescence-activated cell sorting (FACS)

Cells were cultivated in six-well plates for designated duration, followed by centrifugation. The cell pellet was consecutively washed with pre-cooled D-Hanks solution and binding buffer maintained at 4 °C. Subsequently, Annexin V (Thermo Fisher Scientific) and/or propidium iodide (PI) was added, incubating at 37 °C in darkness for 30 min. The FACSCanto II flow cytometer was utilized to determine the cell staining.

### Other cellular functional studies

Caspase-3/-9 activity assay, Trypan blue staining assaying of cell death, and cytosol Cytochrome C ELISA were described in detail in other studies [31–33].

### siRNA

Genechem (Shanghai, China) provided validated siRNAs targeting specific transcription factors (STAT5A, RBPJ, NFYA, NFATC1, WT1, SP1 and STAT1). These siRNAs were applied individually at a concentration of 200 nM for transfection into glioma cells via Lipofectamine 2000. The transfection process was repeated once after 24 h and terminated at 48 h. Each siRNA resulted in a minimum 60-70% reduction in the expression of the targeted mRNA. Control cells were subjected to transfection with a non-sense scramble siRNA (siC, Genechem).

### Chromatin immunoprecipitation (ChIP)

Tissue or cellular lysates underwent homogenization with a homogenizer and were diluted into ChIP buffer provided by Dr. Cao [34]. These prepared lysates were subjected to immunoprecipitation (IP) using an anti-RBPJ antibody. RBPJ-bound DNA was isolated utilizing protein A/G agarose (Santa Cruz Biotech) with NaCl. Following this, quantitative PCR (qPCR) was employed for the precise quantitative analysis of the proposed ORC6 promoter sequence (*GCTGGGAAGG*).

### Animal studies

Xenograft experiments utilized athymic nude mice, aged between 4 and 5 weeks, comprising an equal distribution of male and female subjects, with weights ranging from 17.8 to 18.3 grams. For creating the patient-derived xenograft (PDX) model, P1 glioma cells were directly injected into the brains of nude mice following the described protocol [28, 31, 32, 35]. This led to the development of intracranial P1 glioma xenografts. All animal-related procedures received ethical approval from the Institutional Animal Care and Use Committee (IACUC) and the Animal Ethics Review Board of Shanghai Jiao Tong University.

### Immunohistochemistry (IHC)

The paraffin-embedded tissue sections were subjected to a 60 °C bake for 45 min. Following dewaxing and hydration, the tissue sections underwent treatment with citric acid buffer at 95 °C for 15 min. Subsequent steps included the application of 3% hydrogen peroxide, followed by overnight incubation with the primary antibody at 4 °C. The sections were then heated, rinsed, and subjected to Ki-67 staining (Biyuntian, Wuxi, China) and Ki-67 nuclei ratio was calculated.

### Statistics analysis

Numeric data with normal distribution were represented as mean ± SD (standard deviation). Statistical differences among multiple groups were analyzed using one-way analysis of variance (ANOVA), followed by Scheffe’ and Tukey Test (SPSS 23.0, Chicago, CA). For the comparison between two groups, the significance of variance was evaluated using Student’s t-test (Excel 2007). Statistical significance was defined at a threshold of *P*  <  0.05.

## Results

### ORC6 overexpression in glioma tissues correlates with poor prognosis and various clinical parameters associated with glioma

**Fig. 1:**
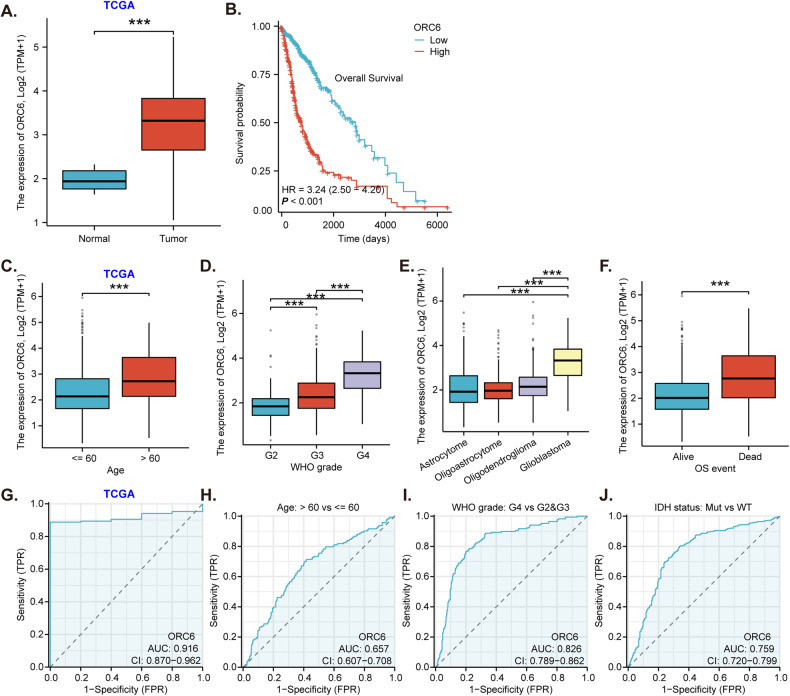
ORC6 overexpression in glioma tissues correlates with poor prognosis and various clinical parameters associated with glioma. TCGA database shows *ORC6* expression in the glioma tissues (“Tumor”) and normal tissues (“Normal”) (**A**). The Kaplan Meier Survival analysis of *ORC6* expression and patients’ overall survival in TCGA-glioma cohort was shown (**B**). *ORC6* expression in the specific glioma patients with described clinical parameters was shown (**C**–**F**). ROC curve analysis of *ORC6* overexpression for a potential biomarker for distinguishing normal and glioma tissues (**G**). The diagnostic values of *ORC6* overexpression in age (**H**), WHO grading (**I**), and IDH status (**J**) of TCGA-glioma patients were shown. “TPM” stands for transcripts per million. “AUC” stands for area under curve. “CI” stands for confidence interval. “HR” stands for hazard rate. *“*TPR” stands for true positive rate. *“*FPR” stands for false positive rate.***P < 0.001.

We first conducted a comprehensive analysis from The Cancer Genome Atlas (TCGA) database to investigate the expression of *ORC6* in glioma. A significantly higher level of *ORC6* mRNA expression in glioma tissues (“Tumor,” *n* = 701) was detected as compared to the normal tissues (“Normal,” *n* = 5) (Fig. 1A). After excluding samples with missing prognosis data (*n* = 3), TCGA glioma samples were categorized into two groups based on the median *ORC6* expression level: 349 samples with high expression and 349 samples with low expression. High *ORC6* expression was associated with poor prognosis in glioma patients (Fig. 1B). Importantly, *ORC6* overexpression showed significant associations with various clinical parameters, including age (Fig. 1C), WHO grading (Fig. 1D), pathological classification (Fig. 1E), and *ORC6* expression within glioma tissues is significantly elevated in deceased patients compared to those who are still alive (Fig. 1F). These results imply that ORC6 overexpression might play a role in different aspects of glioma biology and patient characteristics.

The receiver operating characteristic (ROC) curve analysis for *ORC6* expression yielded an area under the curve (AUC) of 0.916, suggesting that *ORC6* overexpression can be a robust biomarker for diagnosing between normal and glioma tissues (Fig. 1G). The ROC threshold defined for “overexpression” of *ORC6* in Fig. 1G is set at a TPM value of 2.3225. Further analysis revealed that *ORC6* expression also holds diagnostic value in age (Fig. 1H), WHO grading (Fig. 1I), and *IDH* status (Fig. 1J). This indicates its potential as a diagnostic biomarker across various clinical parameters related to glioma. Thus, TCGA database reveals that *ORC6* is significantly elevated in glioma and correlates with poor prognosis and various clinical parameters of glioma.

### ORC6 overexpression in glioma tissues of locally treated patients and different glioma cells

**Fig. 2:**
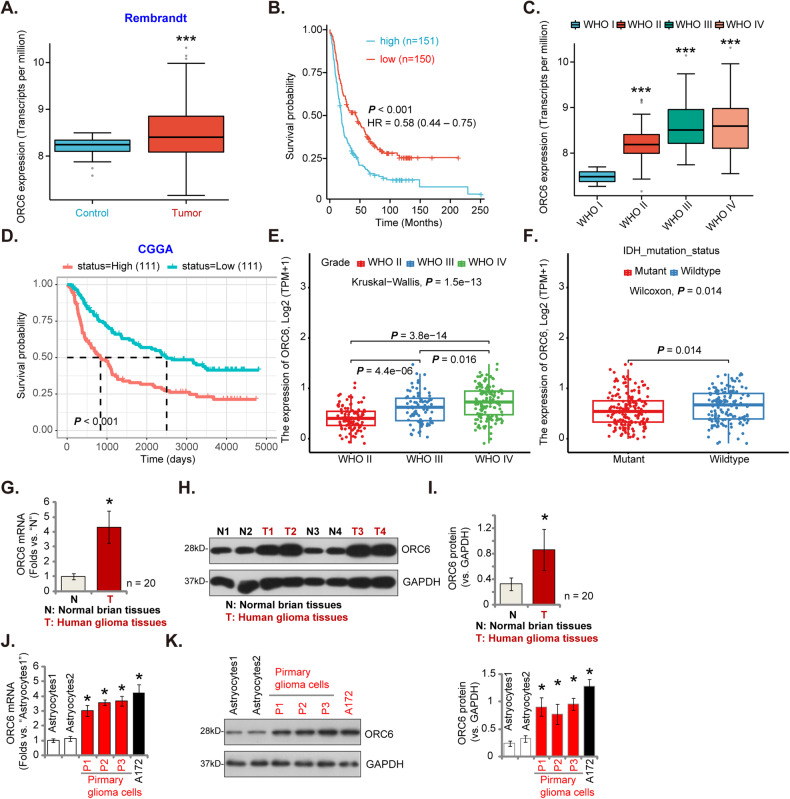
ORC6 overexpression in glioma tissues of locally treated patients and different glioma cells. The expression levels of *ORC6* in glioma tissues compared to normal tissues, sourced from the Rembrandt database, were shown (**A**). The Kaplan-Meier Survival analysis based on *ORC6* expression among glioma patients of the Rembrandt database was shown (**B**). *ORC6* expression in glioma tissues according to different glioma grades from the Rembrandt database was shown (**C**). The Kaplan-Meier Survival analysis derived from the Chinese Glioma Genome Atlas (CGGA) database, illustrating the relationship between *ORC6* expression and survival among glioma patients, was shown (**D**). CGGA database demonstrated the correlation between *ORC6* expression and glioma grade (**E**) or IDH status (**F**) in glioma patients. *ORC6* mRNA and protein expression in glioma tissues (“T”) versus adjacent normal brain tissues (“N”) from twenty (*n* = 20) local high-grade glioma (HGG) patients were shown (**G**–**I**). *ORC6* mRNA and protein expression in primary human astrocytes (“Astrocytes1/2”), immortalized (A172) or primary (“P1,” “P2,” “P3”) glioma cells was presented (**J** and **K**). Data were presented as mean ± standard deviation (SD) (**G**–**K**). *indicate statistical significance (*P* < 0.05) compared to “N” tissues or “Astrocytes1”.

The dataset from Rembrandt on brain cancer encompasses records from 671 patients, sourced from 14 different institutions [36]. As shown, the number of *ORC6* transcripts in glioma tissues (*n* = 454) was significantly higher than that in normal (*n* = 21) brain tissues (Fig. 2A). After removing missing prognosis data, samples were divided equally based on the median *ORC6* expression level (151 *ORC6*-high and 150 *ORC6*-low), an elevation in *ORC6* expression correlates with poor overall survival among glioma patients (Fig. 2B). Additionally, the expression of *ORC6* in high-grade glioma (HGG) significantly exceeds that in low-grade glioma (LGG) tissues (Fig. 2C). Analysis from the Chinese Glioma Genome Atlas (CGGA) database underscores that glioma patients exhibiting heightened *ORC6* expression (*n* = 307) tend to have lower survival rates than those with low *ORC6* expression (*n* = 307) (*P* < 0.001, Fig. 2D). Furthermore, within HGG tissues (grade III–IV), *ORC6* expression is significantly higher compared to that in grade II LGG glioma tissues (Fig. 2E). Importantly, overexpression of *ORC6* is associated with wild-type IDH status within glioma tissues (*P* = 0.014, Fig. 2F), while IDH mutant glioma tissues exhibit comparatively lower *ORC6* expression (Fig. 2F).

Next, the assessment of ORC6 expression was conducted on local human glioma tissues, consisting of twenty (*n* = 20) HGG tissues (“T”) paired with their corresponding adjacent normal brain tissues (“N”) [29, 31–33]. As illustrated in Fig. 2G, the levels of *ORC6* mRNA exhibited a significant increase in local glioma tissues. Furthermore, among four selected HGG patients (Patient-1# to Patient-4#), ORC6 protein expression demonstrated a significant elevation in the glioma tissues (Fig. 2H). Examination of all 20 pairs of ORC6 protein expression data revealed a clear upregulation of ORC6 protein in glioma tissues (*P* < 0.001 vs. “N” tissues, Fig. 2I). Subsequent experiments were carried out to determine whether ORC6 was upregulated in various human glioma cells, including primary human glioma cells (“P1–P3,” derived from three patients [31, 32]) and immortalized A172 cells. The results revealed a significant increase in *ORC6* mRNA expression within the tested glioma cells, compared to that observed in primary human astrocytes (“Astrocytes1/2” [31, 32]) (Fig. 2J). Moreover, the upregulation of ORC6 protein was evident in both primary and immortalized glioma cells (Fig. 2K).

### Exploring the possible functional role of ORC6 in GBM

**Fig. 3:**
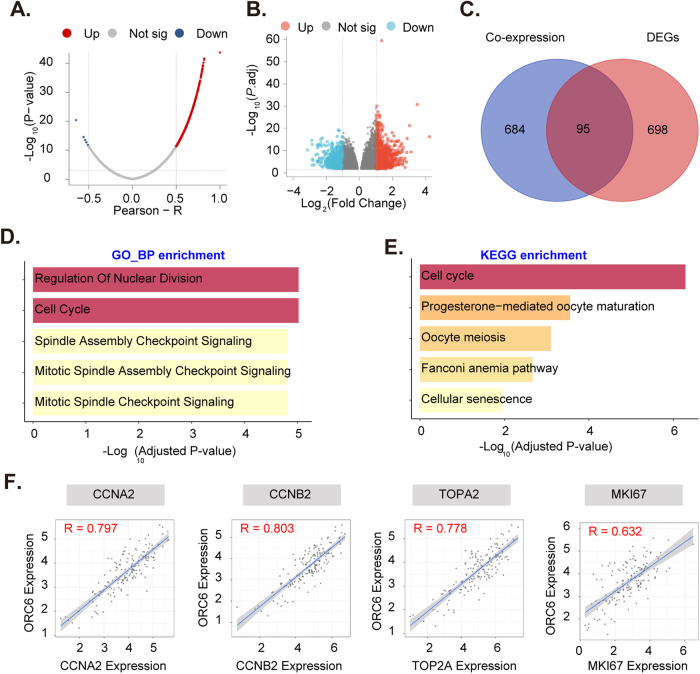
Exploring the possible functional role of ORC6 in GBM. *ORC6* co-expressed genes in TCGA-GBM cohort were shown (**A**). The differentially expressed genes (DEGs) between the high-expression and low-expression groups of *ORC6* in TCGA-GBM cohort were shown (**B**). The Venn diagram illustrated the overlapping genes between *ORC6*’s co-expressed genes and DEGs from TCGA-GBM cohort (**C**). Gene Ontology (GO, **D**) and Kyoto Encyclopedia of Genes and Genomes (KEGG, **E**) analyses of these overlapping genes were presented. The genes with strong positive correlations with *ORC6* expression in TCGA-GBM cohort were shown (**F**).

Through a comprehensive analysis of TCGA-GBM dataset, we explored the possible functional role of ORC6 in GBM. Pearson correlation analysis identified 779 genes significantly associated with *ORC6* (Fig. 3A), while differential expression analysis between high and low *ORC6* expression groups yielded 793 differentially-expressed genes (DEGs) (Fig. 3B). The intersection of these datasets pinpointed 95 common genes linked to *ORC6* in GBM (Fig. 3C). Functional analyses of these common genes using Gene Ontology (GO) predicted *ORC6*’s possible involvement in “Regulation Of Nuclear Division,” “Regulation Of Nuclear Division,” “Spindle Assembly Checkpoint Signaling,” “Mitotic Spindle Assembly Checkpoint Signaling,” among others (Fig. 3D). Additionally, Kyoto Encyclopedia of Genes and Genomes (KEGG) analysis highlighted *ORC6*’s significance in “Cell cycle,” “Cellular senescence,” and other signaling cascades (Fig. 3E). Significantly, *ORC6* demonstrated strong positive correlations with pivotal oncogenic genes, including *CCNA2* and *CCNB2* and *TOP2A* as well as the proliferation marker gene *MKI67* (Fig. 3F). Collectively, these findings underscore *ORC6*’s possible functional role in GBM, implicating its possible involvement in cell cycle dynamics, cell proliferation and mitosis regulation.

### ORC6 silencing or KO results in substantial anti-glioma cell activity

**Fig. 4:**
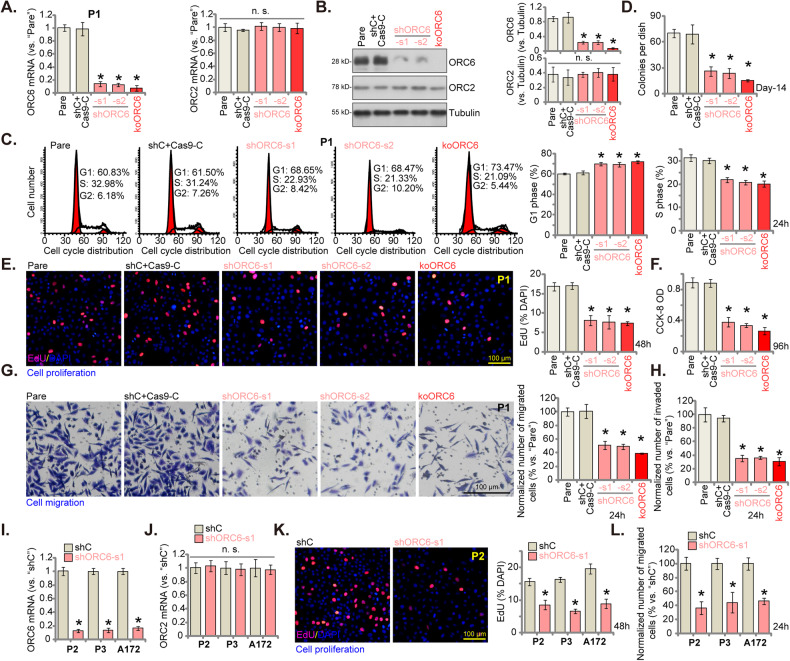
ORC6 silencing or KO results in substantial anti-glioma cell activity. P1 primary glioma cells with the described lentiviral ORC6 shRNA (“shORC6-s1 and shORC6-s2”), the Cas9 construct plus the lentiviral CRISPR/Cas9-ORC6-KO construct (“koORC6”), or the lentiviral scramble control shRNA plus CRISPR/Cas9 control construct (“shC+Cas9-C”), were established, expression levels of the specified mRNAs and proteins were assessed (**A**, **B**). These cells underwent further cultivation for specified durations, followed by analyses of cell cycle distribution, colony formation, proliferation, viability, migration, and invasion using PI-FACS (**C**), clonogenicity (**D**), nuclear EdU staining (**E**), CCK-8 (**F**), “Transwell” (**G**), and “Matrigel Transwell” assays (**H**), respectively. Other primary glioma cells (P2/P3) or established A172 cells were treated with either control lentiviral shRNA (“shRNA”) or ORC6-targeting shRNA (“shORC6-s1”). The expression of *ORC6/2* mRNA was examined (**I**, **J**). These cells underwent further cultivation for specified durations, followed by evaluation of cell proliferation (**K**) and migration (**L**) using similar methodologies. Data were presented as mean ± standard deviation (SD, *n* = 5). The number of migrated or invaded cells was consistently normalized to the total cell count. “Pare” denoted the parental control cells. *P < 0.05 compared to “shC+Cas9-C”/“shC” cells. “n. s.” indicates non-statistical difference (*P* > 0.05). The experiments were conducted five times (biological repeats), yielding consistent results. Scale bar = 100 μm.

We investigated whether reducing ORC6 through genetic methods impacted the behaviors of glioma cells. Initially, we separately introduced two distinct lentiviral shRNAs, shORC6-s1 and shORC6-s2 (with non-overlapping sequences), into P1 primary glioma cells [29, 31–33]. Following puromycin selection, stable glioma cells were established. Alternatively, we utilized a lentiviral CRISPR/Cas9-ORC6-KO construct to establish ORC6 knockout (KO) in P1 glioma cells, termed “koORC6”. When compared to the control P1 glioma cells treated with lentiviral scramble control shRNA plus the CRISPR/Cas9 control construct (“shC+Cas9-C”), the expression of *ORC6* mRNA (Fig. 4A) and protein (Fig. 4B) was significantly reduced in shORC6-s1/s2 P1 glioma cells and koORC6 P1 glioma cells. Importantly, the expression of *ORC2* mRNA and protein, serving as the control, remained unchanged (Fig. 4A, B).

Cell cycle analyses revealed an observed accumulation of cells in the G1 phase and a concurrent decrease in the S phase after silencing or knocking out of ORC6 in P1 glioma cells (Fig. 4C). Moreover, the depletion of ORC6 hindered the proliferation of P1 cells by inhibiting colony formation (Fig. 4D) and reducing the proportion of EdU-positive nuclei (Fig. 4E). Cell viability, tested via CCK-8 assays, was also decreased in ORC6-depleted P1 glioma cells (Fig. 4F). Additionally, genetic silencing or KO of ORC6 impeded the in vitro migration and invasion of P1 glioma cells, as assessed through the “Transwell” (Fig. 4G) and “Matrigel Transwell” (Fig. 4H) assays, respectively. As expected, the treatment with “shC+Cas9-C” did not significantly influence cell cycle progression (Fig. 4C), proliferation (Fig. 4D, E), viability (Fig. 4F), migration (Fig. 4G) or invasion (Fig. 4H) in P1 primary glioma cells.

Subsequently, we investigated whether silencing of ORC6 produced similar effects in other glioma cells, encompassing primary cells derived from other patients (P2 and P3 [29, 31–33]) and immortalized A172 cells. The use of the same shORC6-s1 treatment along with puromycin selection led to a substantial reduction in *ORC6* mRNA across these glioma cells (Fig. 4I), while the expression of *ORC2* mRNA remained unaltered (Fig. 4J). Consequently, the proliferation of glioma cells, as measured by nuclear EdU incorporation, was markedly inhibited in cells expressing shORC6-s1 (Fig. 4K). Additionally, shORC6-s1 inhibited the in vitro migration of these glioma cells (Fig. 4L).

### ORC6 silencing/KO leads to apoptosis activation in glioma cells

**Fig. 5:**
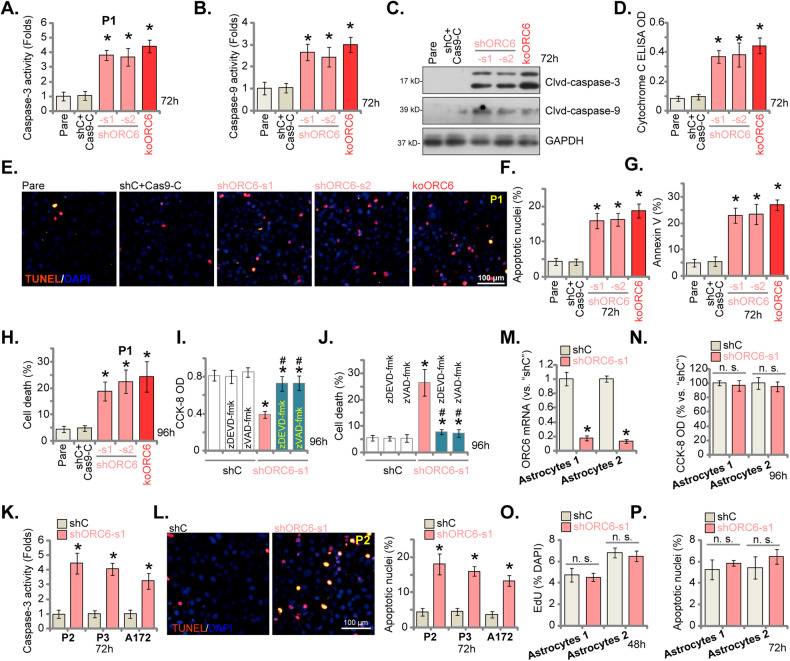
ORC6 silencing/KO leads to apoptosis activation in glioma cells. P1 primary glioma cells with the described lentiviral ORC6 shRNA (“shORC6-s1 and shORC6-s2”), the Cas9 construct plus the lentiviral CRISPR/Cas9-ORC6-KO construct (“koORC6”), or the lentiviral scramble control shRNA plus CRISPR/Cas9 control construct (“shC+Cas9-C”), were established and underwent further cultivation for specified durations; Cytosolic lysates were obtained for the assessment of caspase-3/caspase-9 activity (**A**, **B**), analysis of apoptosis-related protein expression using Western blotting assays (**C**), and quantification of Cytochrome C levels utilizing an ELISA kit (**D**). Cell apoptosis was evaluated using nuclear TUNEL staining (**E**, **F**) and Annexin V FACS (**G**) assays, while cell death was examined through trypan blue staining assays (**H**). P1 glioma cells with control lentiviral shRNA (“shRNA”) or ORC6-targeting shRNA (“shORC6-s1”) were treated with or without the respective caspase inhibitors (each at 50 μM) for specific time periods; Cell viability and death were assessed using CCK-8 (**I**) and Trypan blue staining (**J**) assays, respectively. Other primary glioma cells, P2/P3 or A172 established cells with the lentiviral scramble control shRNA (“shC”) or the lentiviral ORC6 shRNA (“shORC6-s1”) were established and cultured for indicated time periods, the caspase-3 activity (**K**) and apoptosis (nuclear TUNEL staining, **L**) were tested. The primary human astrocytes (“Astrocytes1/2”) were modified to express either “shORC6-Sq1” or “shC,” and expression of *ORC6* mRNA tested (**M**); An equal number of the astrocytes were cultured for designated time periods, cell viability, proliferation and apoptosis were tested by CCK-8 (**N**), nuclear EdU staining (**O**) and TUNEL staining (**P**) assays respectively, with results quantified. Data were presented as mean ± standard deviation (SD, *n* = 5). “Pare” denoted the parental control cells. *P < 0.05 compared to “shC+ Cas9-C”/“shC” cells. “n. s.” indicates non-statistical difference (*P* > 0.05). The experiments were conducted five times (biological repeats), yielding consistent results. Scale bar = 100 μm.

Utilizing “shORC6-s1” and “shORC6-s2” to knock down ORC6 or employing the CRISPR/Cas9 method to KO ORC6 led to increased caspase-3 (Fig. 5A) and caspase-9 (Fig. 5B) activities within P1 glioma cells. Additionally, the levels of cleaved forms of caspase-3 (Clvd-caspase-3) and caspase-9 were elevated in ORC6-silenced/-KO P1 glioma cells (Fig. 5C). Measurement of cytosolic Cytochrome C using an ELISA kit revealed an elevation following ORC6 silencing or KO in P1 glioma cells (Fig. 5D). These results indicated the activation of the mitochondrial apoptosis cascade in ORC6-silenced cells. Apoptosis was observed in shORC6 and koORC6 P1 glioma cells, as evidenced by the increase in the ratio of TUNEL-positive nuclei (Fig. 5E, F). This was corroborated by the heightened Annexin V ratio observed in shORC6 and koORC6 P1 glioma cells (Fig. 5G). The trypan blue staining assay results further indicated that ORC6 silencing or KO led to significant cell death in P1 glioma cells (Fig. 5H). In contrast, the lentiviral scramble control shRNA plus the CRISPR/Cas9 control construct (“shC+Cas9-C”) treatment failed to activate caspase-Cytochrome C pathway (Fig. 5A–D), induce mitochondrial depolarization (Fig. 5E), or trigger apoptosis (Fig. 5F, G), and subsequent cell death (Fig. 5H) in P1 glioma cells.

Crucially, the pan-caspase inhibitor z-VAD-fmk and the caspase-3 inhibitor z-DEVD-fmk significantly mitigated the reduction in P1 glioma cell viability and cell death induced by shORC6-s1, as demonstrated by CCK-8 (Fig. 5I) and Trypan blue staining (Fig. 5J) assays, respectively. In other primary glioma cells (P2/P3) and immortalized A172 cells, the application of shORC6-s1 (as shown in Fig. 4) for silencing ORC6 led to a similar increase in caspase-3 activity (Fig. 5K). Moreover, the heightened percentage of TUNEL-positive nuclei provided further evidence of activated apoptosis in the glioma cells expressing shORC6-s1 (Fig. 5L). Conversely, the silencing of ORC6 via shORC6-s1 in primary human astrocytes (“Astrocytes1/2” [29, 31–33]) (Fig. 5M) did not hinder cell viability (tested by CCK-8 OD, Fig. 5N) and proliferation (tested by nuclear EdU incorporation, Fig. 5O), nor did it induce apoptosis (tested by nuclear TUNEL staining assays, Fig. 5P).

### Overexpression of ORC6 demonstrates pro-tumorigenic activity in glioma cells

**Fig. 6:**
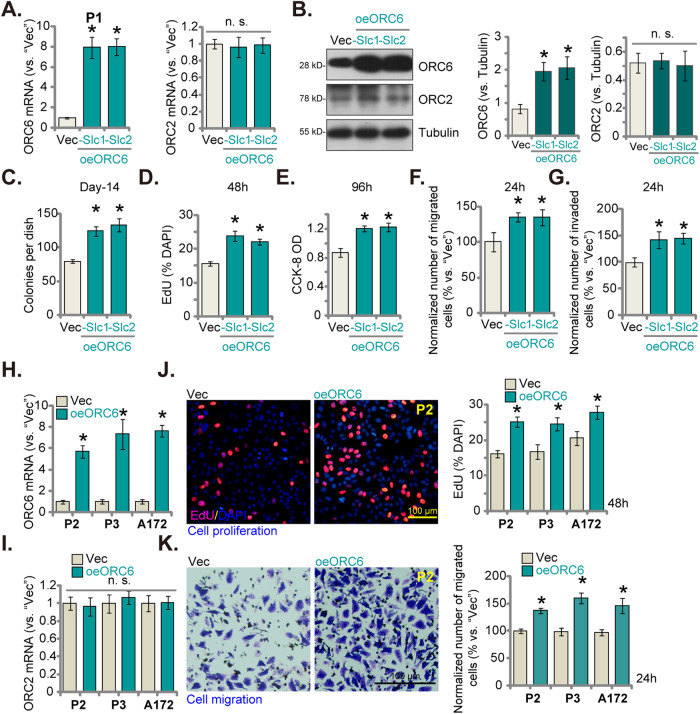
Overexpression of ORC6 demonstrates pro-tumorigenic activity in glioma cells. Two stable P1 glioma cell selections, “oeORC6-Slc1” and “oeORC6-Slc2,” expressing the lentiviral ORC6-expressing construct, were established, along with a control group with the empty vector (“Vec”). Expression levels of the specified mRNAs and proteins were assessed (**A**, **B**); Cells underwent further cultivation for specified durations, followed by analyses of colony formation, proliferation, viability, migration, and invasion using clonogenicity (**C**), nuclear EdU staining (**D**), CCK-8 (**E**), “Transwell” (Figure **F**), and “Matrigel Transwell” assays (**G**), respectively, with results quantified. Other primary glioma cells (P2/P3) or established A172 cells were treated with the lentivirus containing the ORC6-expressing construct (“oeORC6”) or the empty vector (“Vec”). The expression of *ORC6/2* mRNA was examined (**H** and **I**). These cells underwent further cultivation for specified durations, followed by evaluation of cell proliferation (**J**) and migration (**K**) using similar methodologies. The number of migrated or invaded cells was consistently normalized to the total cell count. Data were presented as mean ± standard deviation (SD, *n* = 5). *P < 0.05 compared to “Vec” cells. “n. s.” indicates non-statistical difference (*P* > 0.05). The experiments were conducted five times (biological repeats), yielding consistent results. Scale bar = 100 μm.

The above findings demonstrated a potent anti-cancer effect resulting from ORC6 silencing or KO in different glioma cells. Consequently, we hypothesized that ectopic overexpression of ORC6 might elicit the opposite effect. A lentivirus encoding the ORC6-expressing construct (“oeORC6”) was introduced into P1 primary cells, followed by puromycin treatment to establish two stable selections: “oeORC6-Slc1” and “oeORC6-Slc2”. Subsequent analysis revealed a significant increase in the expression of *ORC6* mRNA (Fig. 6A) and protein (Fig. 6B) in the oeORC6-expressing P1 glioma cells, where the expression of *ORC2* mRNA and protein remained unaltered (Fig. 6A, B). Ectopic overexpression of ORC6 facilitated the proliferation of P1 cells, as evidenced by enhanced colony formation (Fig. 6C) and increased nuclear EdU incorporation ratio (Fig. 6D). Moreover, CCK-8 OD was also augmented in oeORC6 P1 glioma cells (Fig. 6E). The in vitro migration (Fig. 6F) and invasion (Fig. 6G) of P1 glioma cells were accelerated upon ORC6 overexpression. These results further substantiated the pro-cancerous role of ORC6 in glioma cells. The same lentiviral ORC6-expressing construct was employed to generate ORC6-overexpressing cells (“oeORC6”) in additional patient-derived primary human glioma cells (“P2/P3”) and immortalized A172 cells. This resulted in a substantial increase in *ORC6* mRNA expression (Fig. 6H), while the expression of *ORC2* mRNA remained unaltered (Fig. 6I). Remarkably, the oeORC6 glioma cells displayed a significant enhancement in cell proliferation, as indicated by nuclear EdU incorporation (Fig. 6J), along with increase in in vitro cell migration (Fig. 6K).

### ORC6 is important for expression of Cyclin A2, Cyclin B2, and TOP2A within glioma cells

**Fig. 7:**
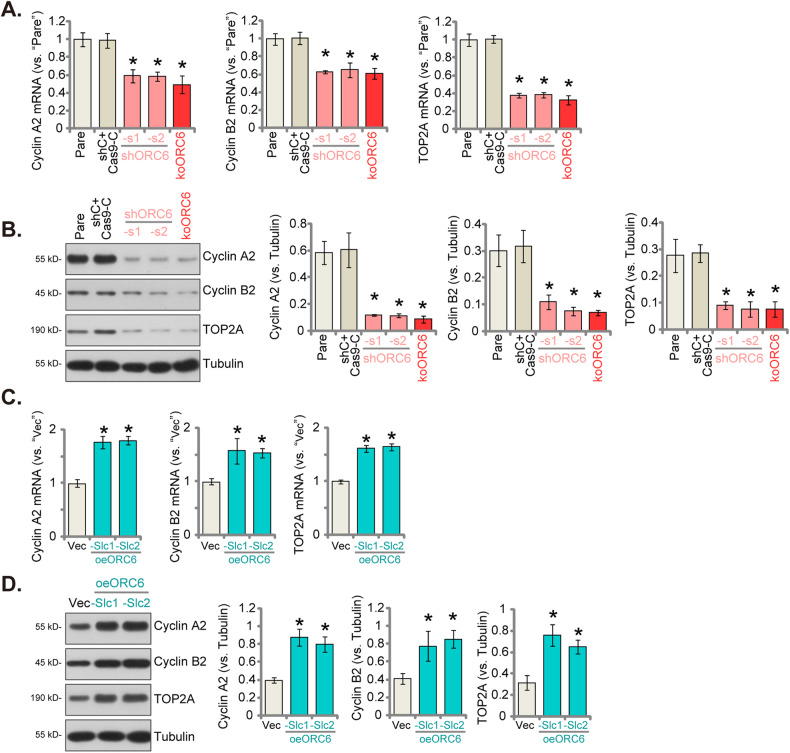
ORC6 is important for expression of Cyclin A2, Cyclin B2, and TOP2A within glioma cells. The P1 primary glioma cells with the described lentiviral ORC6 shRNA (“shORC6-s1 and shORC6-s2”), the Cas9 construct plus the lentiviral CRISPR/Cas9-ORC6-KO construct (“koORC6”), or the lentiviral scramble control shRNA plus CRISPR/Cas9 control construct (“shC+Cas9-C”), were established, expression levels of the specified mRNAs and proteins were assessed (**A**, **B**). Two stable P1 cell selections, “oeORC6-Slc1” and “oeORC6-Slc2,” expressing the lentiviral ORC6-expressing construct, were established, along with a control group with the empty vector (“Vec”). Expression levels of the specified mRNAs and proteins were assessed (**C**, **D**). Data were presented as mean ± standard deviation (SD, *n* = 5). “Pare” denoted the parental control cells. *P < 0.05 compared to “Pare”/“Vec” cells. The experiments were conducted five times (biological repeats), yielding consistent results.

In light of bioinformatic studies highlighting the close association between *ORC6* and key oncogenic genes, including *Cyclin A2*, *Cyclin B2*, and *TOP2A* in GBM (see Fig. 3), our study explored the potential role of ORC6 in regulating their expression in glioma cells. Employing ORC6 silencing using shORC6-s1, shORC6-s2, or CRISPR/Cas9-mediated KO in P1 glioma cells, we observed a significant downregulation in the mRNA expression of these aforementioned genes (Fig. 7A). Furthermore, ORC6 silencing or KO resulted in a corresponding decrease in the protein levels of Cyclin A2, Cyclin B2, and TOP2A (Fig. 7B). In contrast, in P1 glioma cells overexpressing ORC6, “oeORC6-Slc1” and “oeORC6-Slc2,” there was a significant elevation observed in both mRNA and protein expression levels of Cyclin A2, Cyclin B2, and TOP2A (Fig. 7C, D).

### RBPJ functions as a crucial transcription factor of *ORC6* in glioma

**Fig. 8:**
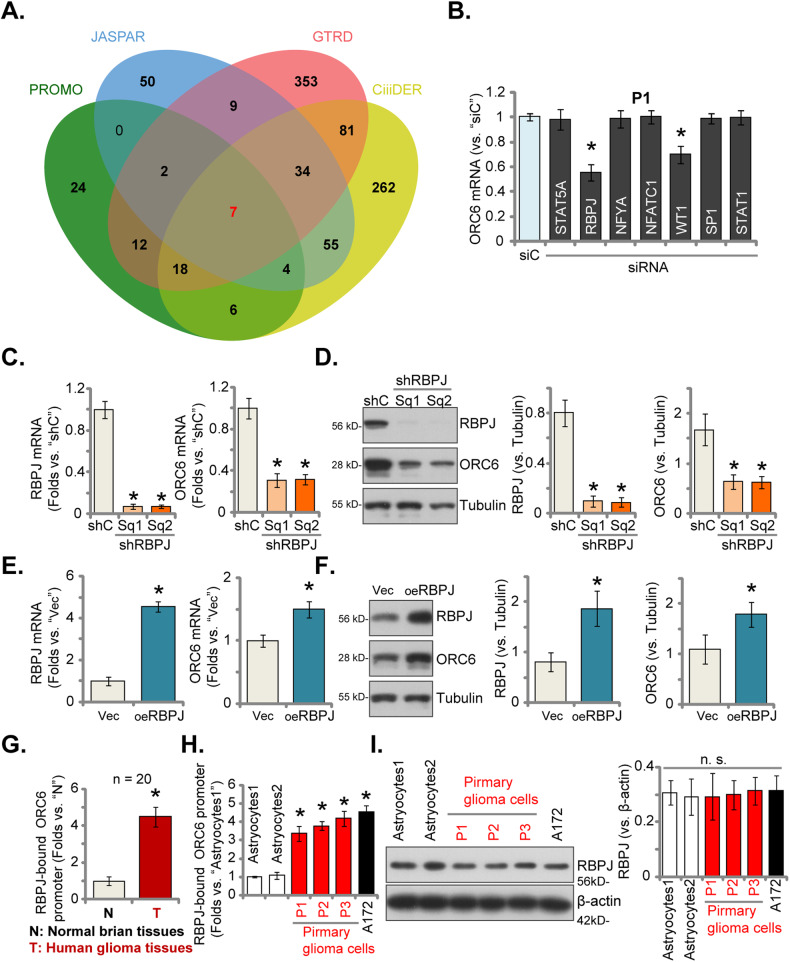
RBPJ is a potential transcription factor of *ORC6* in glioma cells. Multiple databases predicted the common potential transcription factors of *ORC6* (**A**). P1 glioma cells were transfected with the specified siRNAs (at 200 nM) targeting the described transcription factors or a scramble non-sense siRNA (siC, at 200 nM) for 48 h, the expression of *ORC6* mRNA was assessed (**B**). P1 glioma cells were genetically engineered to stably express the lentiviral RBPJ shRNA (shRBPJ-Sq1 or shRBPJ-Sq2) (**C** and **D**), scramble control shRNA (“shC”) (**C**, **D**), the lentiviral RBPJ-expressing construct (oeRBPJ) (**E**, **F**), or an empty vector (“Vec”) (**E**, **F**); The expression of the listed mRNAs and proteins was assessed (**C**–**F**). Chromatin Immunoprecipitation (ChIP) assay results showed the relative levels of RBPJ-bound *ORC6* promoter region in specified glioma tissues (“T”) and matched adjacent normal brain tissues (“N”) (**G**) as well as in listed glioma cells and human astrocytes (**H**). RBPJ protein expression in primary human astrocytes (“Astrocytes1/2”), immortalized (A172) or primary (“P1,” “P2,” “P3”) glioma cells was presented (**I**). Values were mean ± standard deviation (SD). *P < 0.05 versus “siC” (**B**). *P < 0.05 versus “shC”/ “Vec” cells (**C**–**F**). *P < 0.05 versus “N” tissues or “Astryocytes1” (**G**, **H**). “n. s.” indicates non-statistical difference (*P* > 0.05). The experiments were conducted five times (biological repeats), yielding consistent results.

Considering the increase in both mRNA and protein expression of ORC6 within glioma tissues and cells, we next tested the potential involvement of a transcriptional mechanism in driving this upregulation. We first conducted a prediction analysis to identify potential transcription factors (TF) that might bind to the *ORC6* promoter region, spanning from 2,000 base pairs upstream to 100 base pairs downstream of the transcription start point. Four TF databases, PROMO, GTRD, JASPAR, and CiiiDER, were consulted to predict potential TFs of *ORC6*. A total of seven common TFs were identified, including STAT5A, RBPJ, NFYA, NFATC1, WT1, SP1, and STAT1 (Fig. 8A, B). Subsequently, we employed distinct and verified siRNAs targeting the mentioned TFs, individually transfecting them into P1 glioma cells. The results showed that RBPJ siRNA resulted in the most significant decrease in *ORC6* mRNA expression within P1 glioma cells (Fig. 8B). WT1 siRNA also resulted in moderate *ORC6* mRNA downregulation in P1 cells (Fig. 8B), whereas other siRNAs were ineffective (Fig. 8B). These results suggest that RBPJ could be an important TF of *ORC6* in glioma cells.

Subsequently, P1 glioma cells received treatment of lentivirus containing RBPJ shRNA (shRBPJ-Sq1 and shRBPJ-Sq2, using different sequences), leading to the establishment of stable cells after selection. In contrast to P1 glioma cells with scramble control shRNA (“shC”), cells with shRBPJ displayed a remarkable decrease in *RBPJ* mRNA (Fig. 8C) and protein (Fig. 8D) expression, accompanied by a significant reduction in *ORC6* mRNA (Fig. 8C) and protein (Fig. 8D) expression. Contrarily, P1 glioma cells were treated with lentivirus carrying the RBPJ-expressing construct. Through puromycin-based selection, stable cells, “oeRBPJ,” were established. These cells showed a distinct increase in *RBPJ* mRNA (Fig. 8E) and protein (Fig. 8F) levels. Remarkably, oeRBPJ correspondingly led to an increase in *ORC6* mRNA (Fig. 8E) and protein (Fig. 8F) expression in P1 glioma cells.

Significantly, ChIP assay results demonstrated a significant increase in the binding between the RBPJ protein and the presumed *ORC6* promoter region’s binding cites (*GCTGGGAAGG*, from JASPAR database) within glioma tissues obtained from local patients (Fig. 8G). Moreover, this heightened binding was consistently evident across various primary and immortalized glioma cells (P1,P2, P3, and A172) (Fig. 8H). In contrast, RBPJ-*ORC6* promoter binding was relatively weak in normal brain tissues (“N”) (Fig. 8G) and in human astrocytes (Fig. 8H). There was no significant difference in the expression levels of the RBPJ protein between astrocytes and glioma cells (Fig. 8L). These findings strongly suggest that RBPJ functions as a crucial TF for ORC6, and the increased binding between the RBPJ protein and the ORC6 promoter region could potentially be a pivotal mechanism of ORC6 overexpression in glioma.

### ORC6 KO suppresses the growth of intracranial patient-derived glioma xenografts in nude mice

**Fig. 9:**
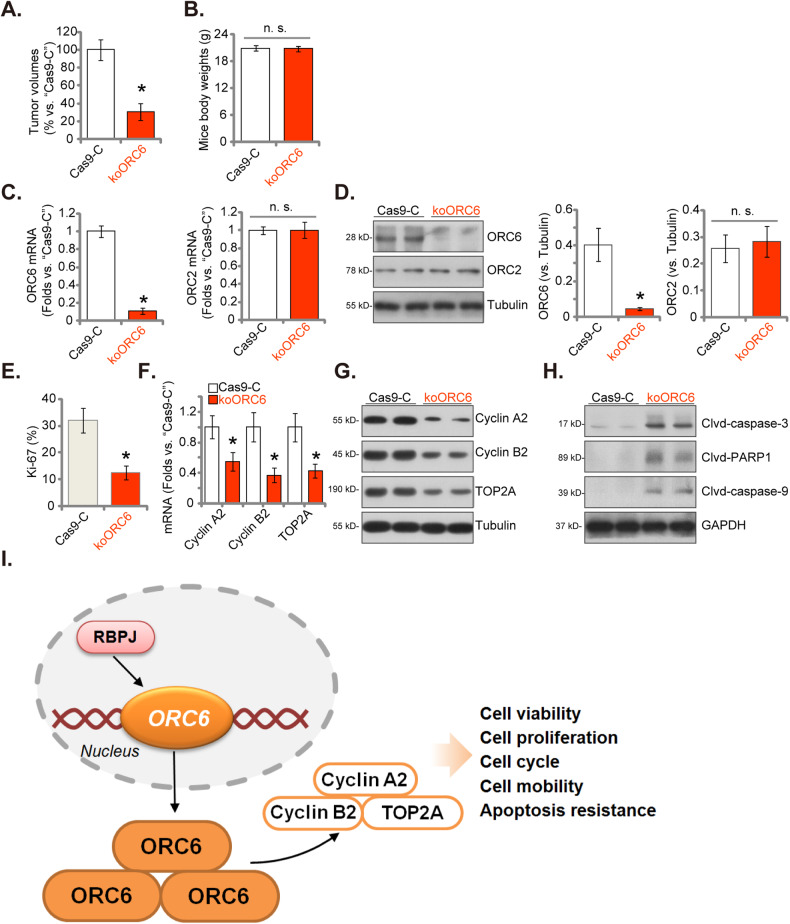
ORC6 KO suppresses the growth of intracranial patient-derived glioma xenografts in nude mice. P1 primary human glioma cells (at 500, 000 cells per mouse), containing either the Cas9 construct with the lentiviral CRISPR/Cas9-ORC6-KO construct (“koORC6”) or the CRISPR/Cas9-control construct (“Cas9-C”), were intracranially administered into nude mice. After a twenty-day period, the intracranial glioma xenografts were analyzed. Tumor volumes (**A**) and the mice’s body weights (**B**) were measured. The specified mRNA and protein levels in the xenograft tissues were assessed (**C**, **D**, **F**–**H**). Additionally, the intracranial P1 glioma xenograft slides underwent immunohistochemistry Ki-67, with results quantified (**E**). The proposed signaling carton of the study (**I**). Data were presented as mean ± standard deviation (SD, *n* = 6). *P < 0.05 compared to “Cas9-C” group. “n. s.” indicates non-statistical difference (*P* > 0.05).

In order to study the potential role of ORC6 in glioma cell growth in vivo, the P1 glioma cells expressing the CRISPR/Cas9-ORC6-KO construct (“koORC6”) or the Cas9-C control (“Cas9-C”) were injected intracranially into the brains of nude mice (according to the previously-described protocols [28, 31, 32]). MRI was utilized to monitor the tumors. After 20 days, the first mouse from the Cas9-C group displayed noticeable symptoms. Subsequently, intracranial xenografts were measured. As shown, the koORC6 intracranial P1 glioma xenografts were significantly smaller in size compared to the Cas9-C intracranial P1 glioma xenografts (Fig. 9A). There were no significant differences observed in the mice’s body weights (Fig. 9B). The mRNA and protein expression of ORC6 robustly decreased in the koORC6 intracranial P1 glioma xenograft tissues (Fig. 9C, D). Conversely, *ORC2* mRNA and protein expression levels remained unchanged (Fig. 9C, D). The quantified Ki-67 IHC staining results revealed a diminished ratio of nuclear Ki67 staining observed in the tissue slides of koORC6 intracranial P1 glioma xenografts, supporting suppressed proliferation (Fig. 9E). Subsequent studies unveiled a significant decrease in the mRNA (Fig. 9F) and protein (Fig. 9G) expression levels of Cyclin A2, Cyclin B2, and TOP2A within koORC6 P1 glioma xenograft tissues, where elevated levels of cleaved-caspase-3, cleaved-caspase-9, and cleaved-PARP-1 were detected (Fig. 9H), indicating apoptosis activation. These collective findings strongly suggest that ORC6 KO impeded the growth of intracranial P1 glioma xenografts in nude mice.

## Discussion

Targeted therapy of glioma concentrates on distinct molecular pathways [9, 37]. Its effectiveness is, however, often hindered by significant drawbacks [9, 37]. Resistance mechanisms can frequently develop against targeted therapies, resulting in treatment failure [9, 37]. Moreover, the blood-brain barrier imposes constraints on drug access to the brain, reducing the efficacy of specific targeted agents [9, 37]. Glioma heterogeneity poses another challenge, as it exhibit diverse genetic alterations, rendering single-target therapies less effective against different tumor subtypes [9, 37]. Therefore, it remains imperative to pinpoint novel molecular targets for glioma.

ORC6, a protein vital for DNA replication and cell cycle progression, primarily facilitates the creation of pre-replication complexes at DNA replication origins. This crucial role ensures the precise and efficient replication of DNA during cell division and cycle progression. Dysregulation of ORC6 has been associated with aggressive tumor behavior and poor patient outcomes, positioning it as a potential biomarker and oncotarget [22–24, 26]. Diminishing ORC6 levels increased the susceptibility of colon cancer cells to 5-FU and cisplatin, multi-nucleation, and activation of p53-associated pathways [22]. ORC6 overexpression significantly correlated with decreased overall survival in clear cell renal cell carcinoma [25].

By employing bioinformatics analyses, we uncovered a substantial elevation in *ORC6* expression within human glioma, correlating with diminished overall survival and disease-free survival, high tumor grade, and wild-type IDH status. Moreover, ORC6 overexpression is evident in glioma tissues derived from locally-treated patients and in a spectrum of primary and established glioma cells. Further bioinformatics investigation unveiled that ORC6 co-expressed genes are enriched in signaling pathways intricately linked to cancer. Depletion of ORC6 using specific shRNA or Cas9-sgRNA KO significantly reduced cell viability and proliferation while disrupting cell cycle progression and mobility, eventually inducing apoptosis in primary and immortalized (A172) glioma cells. Conversely, elevating ORC6 levels via a lentiviral construct intensified malignant behaviors of glioma cells. In vivo experiments demonstrated a significant reduction in the growth of patient-derived glioma xenografts in the mouse brain subsequent to ORC6 KO. Based on the result, it can be concluded that ORC6 represents a promising and new therapeutic oncotarget for human glioma.

Cyclin B2, a key regulatory protein in the cell cycle progression, primarily orchestrates the transition from the G2 phase to the M phase by forming complexes with cyclin-dependent kinases (CDKs), specifically CDK1. In glioma, elevated levels of Cyclin B2 have been associated with glioma progression [38]. Increased transcription and expression of Cyclin B2, dependent on PBK (PDZ-Binding Kinase), emerged as a critical factor in the tumorigenicity and radio-resistance of GBM [39]. Cyclin B2 triggered a senescence-associated secretory phenotype (SASP), increased invasion and augmented proliferation in glioma cells [40]. Cyclin A2 has also been studied in the context of glioma, and its overexpression was associated with poor prognosis in GBM patients [41]. Here, the observed downregulation in both mRNA and protein levels of Cyclin A2 and Cyclin B2 upon silencing or knocking out ORC6, as well as the increased cyclins expression upon ORC6 overexpression in primary human glioma cells (Fig. 9I).

However, the observed alterations in cyclin A2/B2 expression may be a consequence of changes in the cell cycle, rather than factors that influence cell cycle dynamics directly. Thus, the 30-40% decrease in mRNA levels of cyclin A2/B2 following ORC6 silencing or knockout in primary glioma cells, as shown in Fig. 7, could be a secondary effect resulting from the 30-40% reduction in the S phase depicted in Fig. 4. Similarly, the increase in cyclin A2/B2 mRNA levels in glioma cells with ORC6 overexpression (Fig. 7) might also be attributed to an expanded S phase population due to such overexpression. Future research should focus on synchronizing cells with ORC6 manipulation at specific cell cycle phases to directly evaluate the impact on cyclin mRNA levels, independent of broader cell cycle alterations.

Through its enzymatic activity, TOP2A helps DNA unwinding during replication, facilitating the separation of DNA strands [42]. Alterations or increased expression of TOP2A in glioma cells can lead to heightened genomic instability and uncontrolled cell proliferation, often correlating with more aggressive tumor behavior and poor prognoses [43, 44]. TOP2A stimulates glioma cell metastasis possibly by initiating the transcriptional activation of β-catenin [45]. Elevated expression of TOP2A was detected in glioblastoma cancer stem cells (CSCs) and silencing it led to reduced cell proliferation, cell cycle arrest, and increased apoptosis in glioblastoma CSCs [46]. Our data strongly supported that ORC6 regulates the expression of TOP2A within glioma cells. When ORC6 was silenced or knocked out, there was a significant decrease in both mRNA and protein levels of TOP2A. Conversely, overexpression of ORC6 in primary human glioma cells led to an increase in TOP2A expression. TOP2A downregulation was also detected in ORC6 KO intracranial glioma xenograft tissue. Thus, the potential mediation of TOP2A expression could represent another pivotal mechanism underlying ORC6-driven glioma cell growth (Fig. 9I).

Our study explored the potential mechanism responsible for the increased expression of ORC6 in human glioma, highlighting the pivotal role of RBPJ as a crucial transcription factor. RBPJ was shown to promote GBM cell proliferation, invasion, stemness, and tumor initiation via enhancing activation of the IL-6-STAT3 pathway and proneural-mesenchymal transition (PMT) [47]. Xie et al., reported that RBPJ binding to CKD9 promoted transcriptional elongation and maintained brain tumor-initiating cells [48]. Here in P1 glioma cells, experimentally reducing RBPJ expression using lentiviral shRNAs resulted in decreased ORC6 expression at both the mRNA and protein levels. Conversely, elevating RBPJ expression via a lentiviral construct led to increased ORC6 expression. Significantly, our results discovered strengthened binding between the RBPJ protein and the ORC6 promoter region across various glioma tissues and also in primary/immortalized glioma cells. Therefore, enhanced binding between RBPJ-ORC6 promoter might represent a crucial mechanism contributing to the increased expression of ORC6 in human glioma (Fig. 9I). Together, RBPJ-driven ORC6 overexpression promotes glioma cell growth, underscoring its significance as a promising therapeutic target.

### Supplementary information

Figure S1, Original Data

## Data Availability

All data are available upon request.
