# Supplementary figures and images for: Origin recognition complex 6 overexpression promotes growth of glioma cells
Source: Cell Death Dis. 2024 Jul 6;15(7):485. doi: 10.1038/s41419-024-06764-w (PMC11227543; doi:10.1038/s41419-024-06764-w)

Figure S1. The uncropped blotting images of the study.

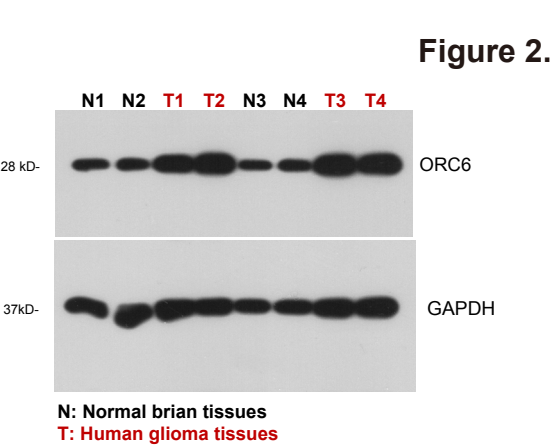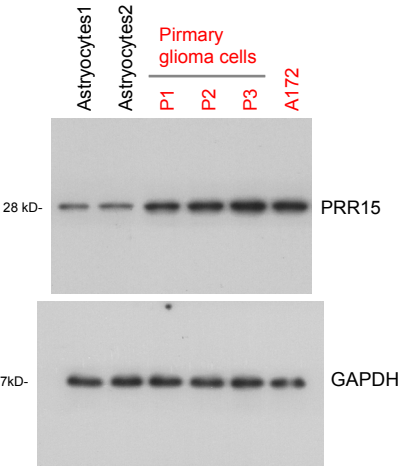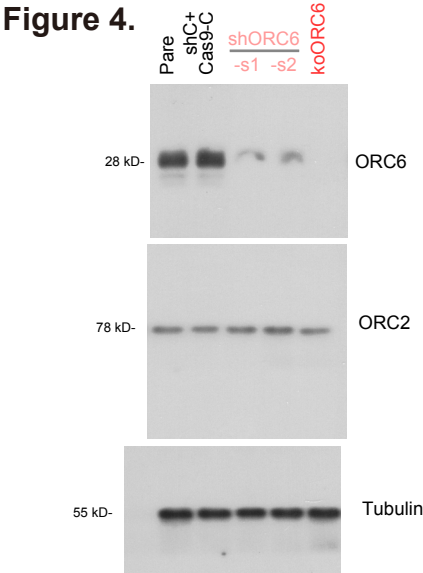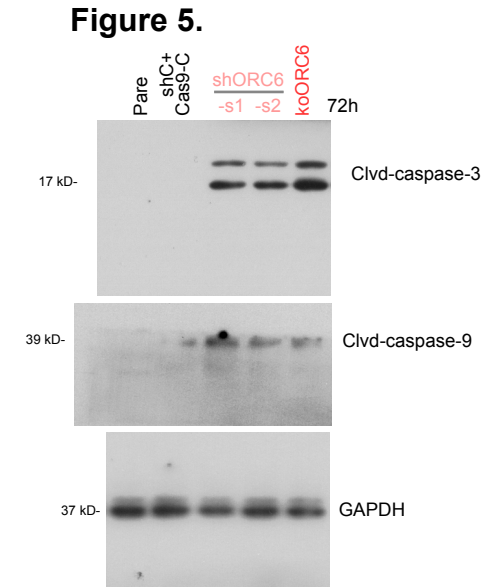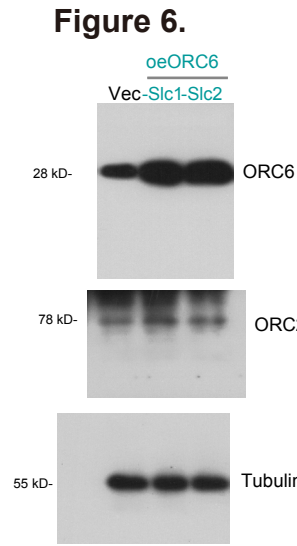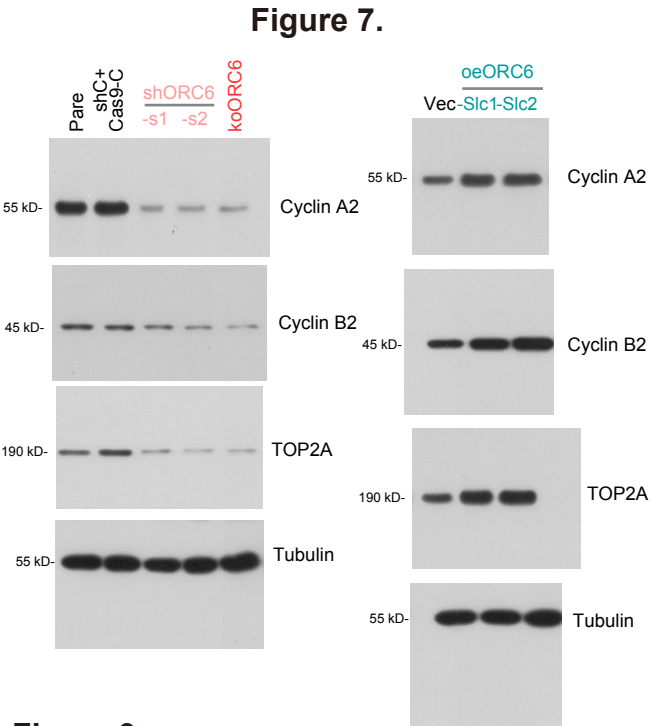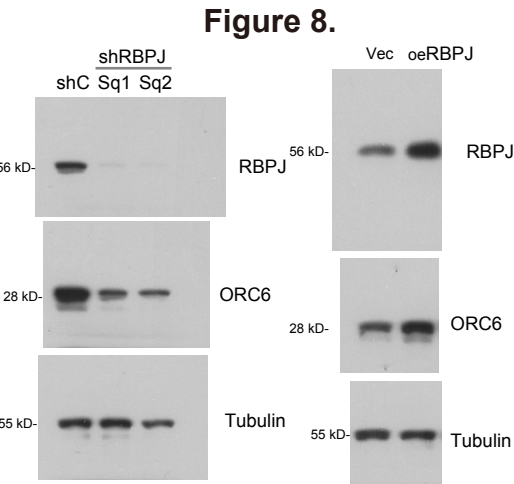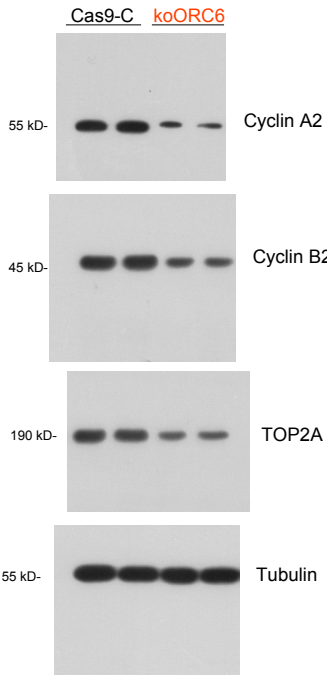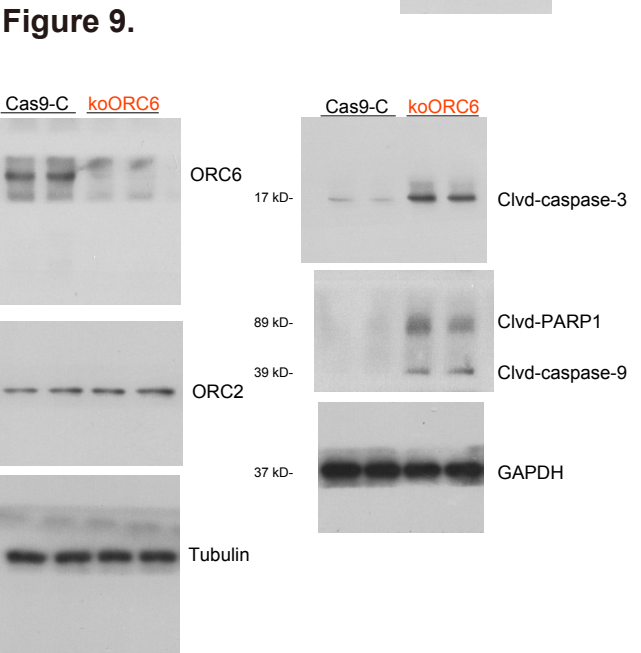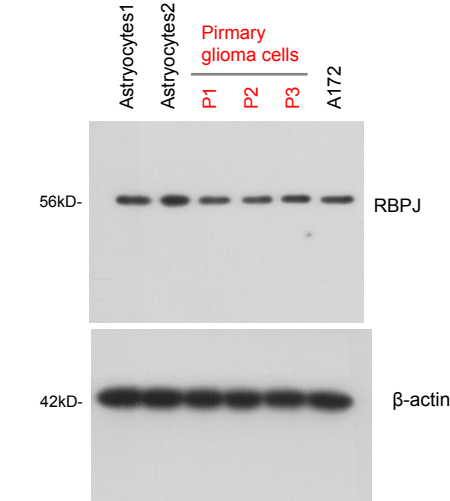

Supplement: Supplementary file 1 — Figure S1, Original Data [file 41419_2024_6764_MOESM1_ESM.pdf]
